# Single center, open label dose escalating trial evaluating once weekly oral ixazomib in ART-suppressed, HIV positive adults and effects on HIV reservoir size in vivo.

**DOI:** 10.1016/j.eclinm.2021.101225

**Published:** 2021-11-29

**Authors:** Nathan W Cummins, Jason Baker, Rana Chakraborty, Patrick G Dean, Enrique Garcia-Rivera, Ashton Krogman, Shaji Kumar, Yury V Kuzmichev, Gregory M Laird, Alan Landay, Mathias Lichterfeld, Maryam Mahmood, Jeffrey Martinson, Mark Maynes, Sekar Natesampillai, Vincent Rajkumar, Yelizaveta Rassadkina, Kristen D. Ritter, Christina G Rivera, Stacey A Rizza, Krupa Subramanian, Aaron J Tande, Elizabeth R Wonderlich, Jennifer A Whitaker, John Zeuli, Andrew D Badley

**Affiliations:** a Division of Infectious Diseases, Mayo Clinic, Rochester, Minnesota, USA; b Division of Infectious Diseases, Hennepin Healthcare, Minneapolis, Minnesota, USA; c Division of Pediatric Infectious Diseases, Mayo Clinic, Rochester, MN; d Department of Surgery, Mayo Clinic, Rochester, Minnesota, USA; e nference, Cambridge, Massachusetts, USA; f Division of Hematology, Mayo Clinic, Rochester, Minnesota, USA; g Department of Infectious Disease Research, Southern Research, Frederick, Maryland, USA; h Accelevir Diagnostics, Baltimore, MD, USA; i Division of Geriatrics, Rush University Medical Center, Chicago, IL, USA; j Ragon Institute of MGH, MIT, and Harvard; Brigham and Women's Hospital, Boston, MA, USA; k Department of Pharmacy, Mayo Clinic, Rochester, Minnesota, USA; l Division of Infectious Diseases, Baylor College of Medicine, Houston, TX, USA; m Department of Molecular Medicine, Mayo Clinic, Rochester, Minnesota, USA

**Keywords:** HIV, Latent reservoir, Proteasome inhibitor, Ixazomib

## Abstract

**Background:**

Achieving a functional or sterilizing cure for HIV will require identification of therapeutic interventions that reduce HIV reservoir size in infected individuals. Proteasome inhibitors, such as ixazomib, impact multiple aspects of HIV biology including latency, transcription initiation, viral replication, and infected cell killing through the HIV protease – Casp8p41 pathway, resulting in latency reversal and reduced measures of HIV reservoir size ex vivo.

**Methods:**

We conducted a phase 1b/2a dose escalating, open label trial of weekly oral ixazomib for 24 weeks in antiretroviral (ART)-suppressed, HIV positive adults (NCT02946047). The study was conducted from March 2017 to August 2019 at two tertiary referral centers in the United States. The primary outcomes were safety and tolerability of oral ixazomib. Secondary outcomes included changes in immunologic markers and estimates of HIV reservoir size after ixazomib treatment.

**Findings:**

Sixteen participants completed the study. Ixazomib up to 4mg weekly was safe and well-tolerated, yielding no treatment-emergent events above grade 1. In exploratory analyses, ixazomib treatment was associated with detectable viremia that was below the lower limit of quantification (LLQ) in 9 participants, and viremia that was above LLQ in 4 of 16 participants. While treatment was associated with reduced CD4 counts [baseline 783 cells/ mm^3^ vs. week-24 724 cells/ mm^3^ p=0.003], there were no changes in markers of cellular activation, exhaustion or inflammation. Total HIV DNA and proviral sequencing were not altered by ixazomib treatment. Intact proviral DNA assay (IPDA) identified intact proviruses in 14 patients pre-treatment, and in 10/14 of those subjects post treatment values were reduced (P=0.068), allowing a calculated intact proviral half life of 0.6 years (95% CI 0.3, 2.5), compared to 7.1 years (95% CI 3.9, 18, p=0.004) in historical controls. Differentiation Quantitative Viral Outgrowth Assays (dQVOA) identified measurable proviruses in 15 subjects pre-treatment; post-treatment values were numerically reduced in 9, but overall differences were not significantly different.

**Interpretation:**

Our study successfully met its primary endpoint of demonstrating the safety of ixazomib for 24 weeks in HIV infected persons. Exploratory analyses suggest that the effects observed ex vivo of latency reversal and reductions in HIV reservoir size, also occur in vivo. Future controlled studies of ixazomib are warranted.

**Funding:**

This study was funded by Millennium Pharmaceuticals Inc..; the Mayo Clinic Foundation; the National Institutes of Health, including the National Institute of Allergy and Infectious Diseases, Division of AIDS, the National Heart, Lung and Blood Institute, the National Institute of Diabetes and Digestive and Kidney Diseases, the National Institute of Neurological Disorders and Stroke, and the National Institute on Drug Abuse. Mayo Clinic also acknowledges generous funding support from Mr. Joseph T. and Mrs. Michele P. Betten.

**Research in context**

**Evidence before this study**

PubMed.gov was queried for terms “HIV”, “reservoir” and “reduction” most recently in October 2021. Multiple approaches are being evaluated to determine if they can be used clinically to reduce the size of the HIV reservoir faster than the slow decline of reservoir size that occurs over time. As there are no treatments yet identified which reduce HIV reservoir size in vivo, and because proteasome inhibitors impact HIV biology including regulation of latency, transcription and infected cell killing, we conducted a phase 1b/2a open label study of oral, once-weekly ixazomib treatment for 24 weeks in HIV positive participants on stable suppressive antiretroviral therapy, using a dose escalating design.

**Added value of this study**

Treatment was safe and well tolerated with no treatment emergent grade 2+ events. In this study, which was not powered to detect treatment emergent differences, exploratory analyses identified that ixazomib was associated with detectable viremia in 13/16 evaluable participants (detectable but below LLQ in 9, detectable and above LLQ in 4) and no change in total HIV DNA as assessed by digital droplet PCR or proviral sequencing. Conversely, ixazomib was associated with non-significant but quantitative reductions in reservoir size as assessed by differentiation Quantitative Viral Outgrowth Assays and Intact Proviral DNA Assays. Calculated proviral half life of subjects on ixazomib using IPDA was 0.6 years (95% CI 0.3, 2.5), versus (7.1 years (95% CI 3.9, 18), p=0.004) in historical controls.

**Implications of all the available evidence**

These data demonstrate safety and tolerability of ixazomib treatment for 24 weeks in HIV positive participants and suggest an anti-reservoir effect which should be further evaluated in controlled clinical studies.

Alt-text: Unlabelled box

## 1 Introduction

The availability of suppressive antiretroviral therapy (ART) now allows for near lifelong control of HIV replication, but yet even prolonged treatment does not cure HIV, since ART discontinuation is nearly universally followed by viral rebound within weeks [1]. The main barrier to HIV cure is the presence of a latent viral reservoir, consisting of replication competent virus within subsets of long lived memory CD4 T cells [[2], [3], [4],^5^]. Intensive efforts to cure HIV have focused on reactivating latent virus, in order that virus induced cell death may result, and/or immune mechanisms will kill residual HIV infected cells, thereby reducing HIV reservoir size [6]. To date these approaches have faltered due to either lack of effectiveness of latency reversal *in vivo*, or lack of reduction in the latent viral reservoir despite induction of viral replication [7], [8], [9], [10].

As obligate intracellular parasites, viruses co-opt host cellular machinery to achieve their evolutionary goals of ensuring survival of infected cells and maximizing virus replication, thereby allowing virus spread to other cells or hosts. Best characterized of these virus host interactions are the dependence of viruses on host nucleic acid replicative machinery, transcription factors and nucleotides. In addition, viruses have also evolved to co-opt other host pathways, for example those governing post translational modifications including the ubiquitin proteasome system. Protein ubiquitination is ubiquitous, and impacts cellular and viral proteins in multiple ways; for example ubiquitination can target proteins for degradation via the proteasome, alter their cellular location, affect their activity, and promote or prevent protein interactions. Viral protein interactions with the ubiquitin system can impact viral entry and intracellular trafficking, transcription, latency and viral cytopathic effect, cell cycle regulation, inhibition of infected cell apoptosis, cellular proliferation, and virus budding (reviewed in [11]). The cellular ubiquitin proteasome system regulates a number of steps in the HIV replication cycle [12], including latency [13], through NF-kB dependent activation of viral transcription [14]; promotion of viral transcription and elongation through stabilization of HSP90-pTEFb complex [15]; or by enabling Tat-transactivation of viral transcription [13].

At its most fundamental level, HIV infection causes a disease characterized by disordered cell death. During uncontrolled infection, there is excessive death of predominantly uninfected immune cells causing immunodeficiency that leads to infected individuals becoming susceptible to opportunistic infections and malignancies. Conversely, during treated HIV infection, a subset of infected cells, which contains replication competent HIV, are resistant to cell death, even upon viral reactivation, which is the main obstacle to achieving a cure for HIV infection. Much recent interest has focused on developing pharmacologic strategies of reactivation, which promote the death of the reactivating cell.

HIV infection of an individual T cell most often results in cell death through a number of proposed pathways, including pyroptosis, innate sensing of HIV proteins or nucleic acids, or apoptosis [16]. One pathway of HIV induced cell death occurs at a post integration step, and involves HIV protease which is active within the infected cell cytosol, cleaving both the HIV polyprotein as well as host proteins including Procaspase 8 [17]. The HIV specific caspase 8 fragment, called Casp8p41, adopts an alpha helical conformation which resembles a BH3 like conformation normally associated with the Bcl2 family of apoptosis regulatory proteins [18]. Casp8p41 then can bind with nanomolar affinity to the proapoptotic mitochondrial protein Bak, causing it to oligomerize, activate and allow release of proapoptotic Cytochrome c into the cytosol, which activates the effector phase of apoptosis. The interaction of Casp8p41 with Bak occurs through the BH3-like domain of Casp8p41 binding to the BH3 groove in Bak, and can be abrogated by mutagenesis [18]. Not surprisingly the BH3-like domain of Casp8p41 can also bind proteins which also contain a BH3 groove, such as Bcl2, with similar nanomolar affinity [19]. When Casp8p41 binds Bcl2, cell death does not occur, and the Casp8p41:Bcl2 complex is ubiquitinated and degraded by the proteasome [14]. Inhibiting the degradation of this complex using proteasome inhibitors, results in activation of the unfolded protein response [20], and induction of cell death with characteristics of apoptosis [14,20].

Proteasome inhibitors are approved treatments for plasma cell malignancies including myeloma, where they improve both progression free and overall survival of patients afflicted with these disorders. Multiple myeloma cells produce excessive amounts of IgG, which is compensated intracellularly by the microautophagy and the ubiquitin proteasome pathway removing excess intracellular immunoglobulins. Blockade of the proteasome by proteasome inhibitors results in accumulation of intracellular immunoglobulin, induction of the unfolded protein response, leading to autophagic cell death [21].

Proteasome inhibitors have also been studied in the setting of HIV. HIV Env is ubiquitinated and degraded by the proteasome, and proteasome inhibition increases Env [22] and interferes with Gag polyprotein processing [23], although these studies did not evaluate whether a functional consequence of this is death of the HIV infected cells. Accumulating data also indicate that proteasome inhibitors act as latency reversal agents, first described with bortezomib reactivating HIV in a Bcl2-dependent latency model [24]. Others have since confirmed that the proteasome inhibitors bortezomib and carfilzomib reactivate latent HIV *in vitro* [13,15,24,25].

Ixazomib is an oral, once weekly proteasome inhibitor US Food and Drug Administration (FDA) approved for use in patients with multiple myeloma, with a favorable safety profile. Using HIV long terminal repeat (LTR) reporter constructs as well as primary CD4 cells isolated from HIV infected subjects we showed that ixazomib induces HIV reactivation in both cell lines and in patient cells *ex vivo,* and that reactivation induced by ixazomib results in selective killing of reactivated, HIV-infected cells in a Casp8p41-dependent mechanism leading to quantitative reductions in reservoir size as measured by total and integrated HIV DNA [14]. Based on the ability of ixazomib to reactivate cells from latency, as well as its *ex vivo* effect of reducing reservoir size, we conducted a Phase 1b/2a open label clinical trial of ixazomib in ART-suppressed, HIV-positive persons (Clinicaltrials.gov NCT02946047), to evaluate the safety and tolerability of oral ixazomib in ART-suppressed, HIV-positive persons, and to explore whether treatment induces detectable HIV viremia in ART suppressed patients, and/or results in changes in HIV reservoir size..

## 2 Methods

### 2.1 Trial Design and Oversight

We conducted a Phase 1a/2b open label, uncontrolled, dose escalation trial of oral ixazomib in antiretroviral suppressed, HIV positive adults. The trial was registered with ClinicalTrials.gov (NCT02946047) and was approved by the Mayo Clinic Institutional Review Board (IRB#16-001938) and conducted in accordance with all federal, local and institutional guidelines. The investigational use of ixazomib was approved under an Investigational New Drug application (IND-130299) of the United States Food and Drug Administration (FDA), and the trial design and lack of untreated control group determined by the FDA as part of the IND application. The trial was funded by Takeda Pharmaceuticals. The trial was monitored by an independent Data Safety Monitoring Board (DSMB). Reporting of the trial adheres to CONSORT guidelines.

Participants were recruited from two sites, Mayo Clinic, Rochester, MN and Hennepin Healthcare, Minneapolis, MN. Written informed consent was obtained from all patients. Participants were eligible for participation if they were ≥ 18 years of age; HIV positive with suppressed viral replication on at least 3 active antiretrovirals for at least 6 months, with a plasma viral load <20 copies/ ml within 30 days of enrollment; and a CD4 count >500 cells/ mm^3^ within 30 days prior to study enrollment. A full list of exclusion criteria can be found in the complete study protocol (Appendix 1), and included significant baseline laboratory abnormalities; recent or ongoing infections other than HIV, malignancy or surgery; women of childbearing potential, pregnant or nursing women; history of peripheral neuropathy or evidence of peripheral neuropathy on screening; uncontrolled cardiovascular conditions or baseline QTc >450 milliseconds; systemic treatment with strong CYP3A inducers; known history of liver disease, cirrhosis, active hepatitis B or hepatitis C infection.

### 2.2 Treatment and Procedures

Ixazomib dose escalation for the study groups is outlined in Figure S1. Ixazomib was taken orally once weekly on days 1, 8, and 15 of a 28-day cycle for the first 12 weeks (cycles 1-3). During cycles 1-3, no dose was administered on day 21, mirroring current FDA-approved dosing structure for multiple myeloma. An independent DSMB reviewed cohort safety data after 12 weeks to determine whether it was safe to increase 4 doses (4th dose administered on day 21) per 28 days for the second 12 weeks (cycles 4-6). Subsequent initiation of escalated dose cohorts began after DSMB review of safety data after 28 days of treatment from the preceding lower dose cohort. The ixazomib drug product (the active boronic acid) was provided in strengths of 4.0-, 3.0-, and 2.0-, and 0.5-mg capsules to attain the respective dose for each dosing cohort. The first 4 participants received a 1mg dose (A cohort); 3 participants received a 2mg dose (B cohort); 3 participants received a 3mg dose (C cohort); and 7 participants received a 4mg dose (D and E cohorts). Plasma HIV viral load testing was performed using the cobas HIV-1 - Quantitative nucleic acid test for use on the cobas 6800/8800 Systems (Roche Molecular Systems, Inc., Branchburg, NJ).

### 2.3 Outcome measures

The primary outcomes in the study were the safety and tolerability of ixazomib in HIV positive patients on antiretroviral therapy. Pre-specified secondary outcomes included change from baseline in measures of HIV infection (total cell associated HIV DNA by digital droplet PCR and culturable HIV by quantitative viral outgrowth assay), and immune parameters (CD4 and CD8 T cell counts, as well as CD4/CD8 Ratio).

### 2.4 Total cell associated HIV DNA by digital droplet PCR

Total genomic DNA (gDNA) was isolated from 5 to 10 million CD4 cells using DNeasy Blood & Tissue Kit (Qiagen) according to manufacturer's protocol. Genomic DNA was restriction digested using EcoRI. The ddPCR mix was made by adding 10 µl of sample containing gDNA, 900 nM of Primers and 300 nM of probe (Integrated DNA Technologies) with 10 µl of 2x ddPCR supermix for probes (Bio-Rad), in a final volume of 20 µl. Automated ddPCR, the reaction was prepared as described above with samples and controls in a 96-well PCR plate, and generated droplet using automated droplet generator (AutoDG). Following completion, the plate was heat sealed, amplified using PCR protocol consisted of initial denaturation at 95 °C for 10 min, followed by 45 cycles of 95 C for 30 s denaturation and annealing/elongation at 60 C for 1 min with a ramp rate of 2.5 °C/s, and final step of 98 °C for 10 min to deactivate enzyme. Each sample was measured in quadruplicates. Droplets were read by the QX200 droplet reader (Bio-Rad) and the results from the QX200 droplet reader were recorded in Bio-Rad's QuantaSoft Analysis Pro software using absolute quantification. The software counts the number of positive and negative droplets and uses Poisson statistics to calculate copies/μL. A manual threshold was applied based on positive and non-target control samples in each experiment to accurately separate positive and negative clusters and eliminate false positive droplets [26].

**Table:** 

HIV pol forward (mf299)	GCA CTT TAA ATT TTC CCA TTA GTC CTA
HIV pol reverse (mf302)	CAA ATT TCT ACT AAT GCT TTT ATT TTT TCTTC
HIV pol probe (mf348)	FAM-AAG CCA GGA ATG GAT GG-MGB
RPP30 forward	GAT TTG GAC CTG CGA GCG
RPP30 reverse	GCG GCT GTC TCC ACA AGTC
RPP30 probe	VIC-CTG ACC TGA AGG CTC T-MGB

### 2.5 Intact Proviral DNA Assay (IPDA)

In-depth descriptions of the IPDA design and procedure have been previously published (cite PMIDs 30700913, 32690683). In the present study, the IPDA was performed by Accelevir Diagnostics under company standard operating procedures. The assay was performed on genomic DNA from CD4+ T cells isolated from cryopreserved PBMCs. Operators were blinded to donor identity and time point.

### 2.6 Replication competent HIV-1 reservoir quantification

Baseline and post-treatment HIV-1 reservoir measurements were generated through the quantitative viral outgrowth assay coupled with ex vivo memory differentiation (dQVOA) as described previously [27]. To minimize dQVOA testing batch effects, samples from each study arm were distributed across each batch of assays performed. Briefly, cryopreserved peripheral blood mononuclear cells (PBMC) were reanimated, rested overnight, and magnetically enriched for resting CD4+ T cells (rCD4) (StemCell, 17962 and 19250). Immediately after magnetic enrichment and before ex vivo differentiation, rCD4 were plated at concentrations of 5 × 10^5^, 2 × 10^5^, 4 × 10^4^, and 8 × 10^3^ cells per well in complete media with 25 ng/mL recombinant human TNF-α, IL-6, IL-7, IL-10, and IL-15 (R&D Systems, 210-TA, 206-IL, 207-IL, 217-IL, 247-ILB). After 7 days of ex vivo differentiation and without pooling, splitting or cross-contaminating, differentiated cultures were stimulated with 0.5 µg/mL purified PHA (ThermoFisher Scientific, Remel, R30852801), 100 IU/mL recombinant human IL-2 (R&D Systems, 202-IL), and a mixture of γ-irradiated PBMC from at least three allogeneic HIV-naïve donors at a ratio of 10:1. Cultures were maintained for an additional 11 days. Appropriate media and cytokine replenishments occurred every 3-4 days and extent of magnetic enrichment and memory differentiation were monitored by standard flow cytometric methods. Each dQVOA well was scored as positive or negative for HIV-1 reactivation and outgrowth by quantifying supernatants for HIV-1 p24 antigen by enzyme linked immunosorbent assay (ELISA) (PerkinElmer, NEK050B001KT). Supernatants containing at least 3.25 pg/mL of HIV-1 p24 antigen were scored as positive and reservoir sizes were calculated using IUPMStats v1.0 [28].

### 2.7 HIV-1 Near Full-Length Sequencing

Genomic DNA was extracted from the total PBMCs using the DNeasy Blood and Tissue Kit (Qiagen). The DNA was then diluted to single genome levels based on Poisson distribution statistics. Droplet digital PCR results were subjected to single-genome amplification using Invitrogen Platinum Taq and nested primers spanning near full-length HIV-1 (HXB2 coordinates 638-9632), as describe previously [29]. PCR products were then visualized by agarose gel electrophoresis (ChemiDoc MP Image Lab, BioRad). All near full-length and/or 5-amplicon positive amplicons were subjected to Illumina MiSeq sequencing at the Massachusetts General Hospital (MGH) DNA Core facility. Resulting short reads were de novo assembled using Ultracycler v1.0 and aligned to HXB2 to identify large deleterious deletions (<8000bp of the amplicon aligned to HXB2), out-of-frame indels, premature/lethal stop codons, internal inversions, or packaging signal deletions (≥15 bp insertions and/or deletions relative to HXB2), using an automated in-house pipeline written in Python programming language (https://github.com/BWH-LichterfeldLab/Intactness-Pipeline) [30], consistent with prior studies [29,31,32]. Presence/absence of APOBEC-3G/3F-associated hypermutations was determined using the Los Alamos National Laboratory (LANL) HIV Sequence Database Hypermut 2.0 program [33]. Viral sequences were categorized as “genome-intact” if they lacked all the mutations listed above. The alignment of the sequences was then performed using MUSCLE [34]. Phylogenetic distances between sequences were examined using maximum likelihood trees in MEGA-X (www.megasoftware.net). Visualization of the sequences was then performed using Highlighter plots (https://www.hiv.lanl.gov/content/sequence/HIGHLIGHT/highlighter_top.html). Viral sequences were considered clonal if they had completely identical consensus sequences; single nucleotide variations in primer binding sites were not considered for clonality analysis. All graphs and pie charts were created using Prism v.8 (GraphPad).

### 2.8 Flow Cytometry for Activation and Exhaustion

Phenotypic analyses were performed to a determine immune activation, immune checkpoint, and markers of cell proliferation in T lymphocyte subsets. In brief, frozen PBMC from HIV positive subjects (Visit 2 and Visit 13) were thawed rapidly in a 37°C water bath, transferred into RPMI 10% FBS complete media, collected by centrifugation, washed in PBS, stained for cell viability, washed in PBS, and stained for cell surface markers. The PBMC were then washed in FACS Buffer (PBS-0.5% BSA + 1% Na Azide) and stained for intracellular Ki67 using the BD Cytofix\Cytoperm kit, washed 2X in perm\wash buffer, resuspended in 1% paraformaldehyde and held refrigerated until analyzed by flow cytometry. The following reagents were used for staining: Live/ Dead Fixable Aqua Dead Cell Stain Kit [Invitrogen, Waltham, MA], mouse anti-human CD3 APC-R700 [BD Biosciences, San Diego, CA/Clone UCHT1), mouse anti-human CD4 PE-CF594 [BD, Clone RPS-T4], mouse anti-human CD8 APC-H7 [BD, Clone SK1], mouse anti-human CD38 BV711 [BD, Clone HIT2], mouse anti-human HLA DR BV421 [BD, Clone G46-6], mouse anti-human CD71 [BD, Clone M-A712], mouse anti-human CD73 PerCP-Cy5.5 [BD, Clone AD2], mouse anti-human CD160 AF488 [BD, Clone BY55], mouse anti-human LAG-3 PE [BD, Clone 7D3], mouse anti-human PD-1 PE-Cy [BD, Clone EH12.1], mouse anti-human TIM-3 BV650 [BD, Clone T47-530], and mouse anti-human TIGIT APC [RnD Systems, Clone 741182]. The following antibodies were used for intracellular experiments Ki67 BV786 (BD, Clone B56) and mouse IgG1 BV786 (BD, Clone X40). Flow cytometry was performed using a BD LSRFortessa™ SORP flow cytometer and results were analyzed with FlowJo v9.9.6 software (Tree Star, Inc).

### 2.9 Multiplex cytokine array

32 plasma samples, from Visit 1 and Visit 7, were tested for cytokine/chemokine/Growth factors. These samples were run at single concentrations without any dilutions, based around the manufacturer's protocol suggested concentrations. GM-CSF, IFNα2, IFNγ, IL-1α, IL-1β, IL-1RA, IL-2, IL-3, IL-4, IL-5, IL-6, IL-7, IL-8/CXCL8, IL-9, IL-10, IL-12(p40), IL-12(p70), IL-13, IL-15, IL-17A/CTLA8, IP-10/CXCL10, MCP-1/CCL2, MIP-1α/CCL3, MIP-1β/CCL4, TGFα, TNFα, TNFβ/Lymphotoxin-a levels were determined using MagPix™ Milliplex (Luminex® technology). Luminex technology uses color-code microspheres with fluorescent dyes which are coated with a specific capture antibody. After incubation of the analyte with captured beads, a biotinylated detection antibody is introduced followed by Streptavidin-PE conjugate, the reporter molecule. The data were acquired using the Luminex analyzer (MagPix) and each individual microsphere were identified, and the assay is quantified based on fluorescent reporter signal. Cytokine production was calculated using Median Fluorescent Intensity (MFI). The best-fit standard curve was determined by regression analysis using five-parameter logistic curve-fit (5-PL) as suggested in product manual for calculating analyte concentrations in samples. Duplicates of each concentration were run, so that coefficient of variation (CV) values could also be evaluated for the panel. These measurements were performed by The Cytokine Core, LLC (Indianapolis, IN)

### 2.10 Statistical Analysis

Descriptive statistics are used to summarize patient characteristics, event rates and outcome variables, including numbers (percentages), medians (interquartile ranges). Intention to treat analyses are presented for the primary endpoint of safety. Per protocol analyses are presented for secondary endpoints. Nonparametric statistical tests for comparisons when used are detailed in the text and figure legends, with paired tests used for pre- and post- treatment comparisons within individuals. P values <0.05 are considered statistically significant. Dunn's methods for corrections for multiple comparisons were used as appropriate (Fig S2 and Fig S3).

### 2.11 Role of the Funding Sources

The funding institutions played no role in trial conduction, data analysis or determining the reporting of these results.

## 3 Results

**Fig. 1:**
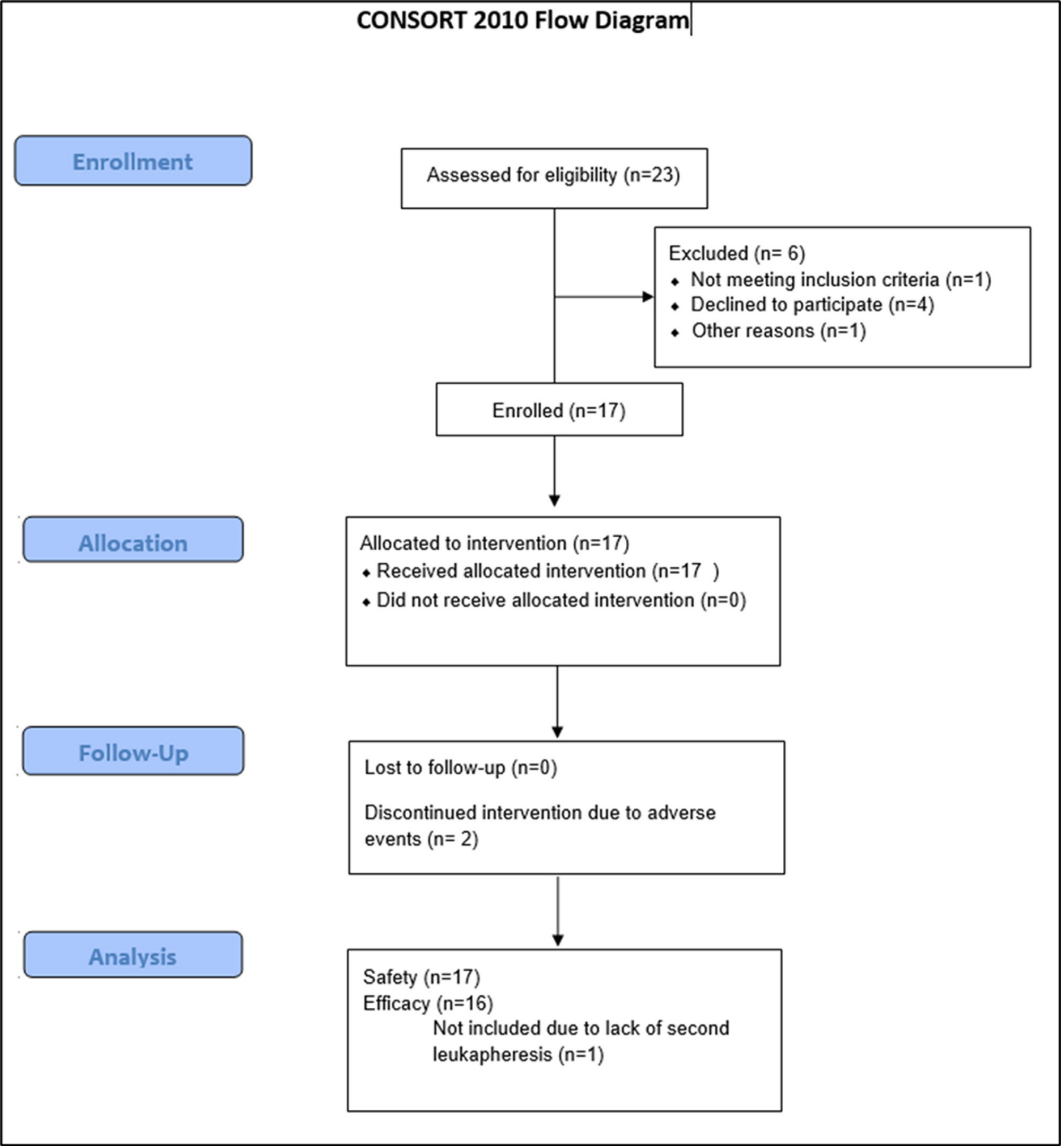
CONSORT Flow diagram detailing participant screening, enrollment, follow up and analysis.

Seventeen HIV positive persons with prolonged viral suppression (median duration of virologic suppression prior to study entry was 2.6 years (interquartile range [IQR] 1.2, 4.3)) on ART were enrolled (Fig. 1; Supplementary Table S1). Sixteen (94%) of the participants were male. Median duration of virologic suppression prior to study entry was 2.6 years (IQR 1.2, 4.3). ART regimen backbones were based on integrase inhibitors (N=13), non-nucleoside reverse transcriptase inhibitors (N=2), and combination protease-inhibitor plus integrase inhibitor (N=2).

Participants were treated with oral ixazomib for 24 weeks, in a dose-escalation cohort design, while continuing ART (Fig. S1). The prespecified primary endpoint for this study was safety and tolerability, and 16 (94%) participants completed the study protocol, while 15 (88%) participants completed all 24 weeks of study treatment. One participant in the 1mg group met predefined stopping criteria after the third dose, because of a drop in absolute CD4 T cell count to below 350 cells/ mm^3^, which was associated with acute infection with Human Herpes Virus-6 deemed by DSMB review to be unrelated to study treatment. One participant in the 4mg group stopped study medication after 20 weeks due to maculopapular rash but completed the post treatment leukapheresis. No Grade 2 or worse adverse events (AEs) occurred during follow-up of any participants. The most common adverse events included diarrhea (17.6%), which occurred early and was self-limited, and mild, maculopapular rash (11.8%). One participant in the 3 mg group developed transient symptoms concerning for peripheral neuropathy, but that on further clinical evaluation was deemed unrelated to study medication and did not result in study discontinuation. A summary of reported adverse events can be found in Supplementary Table S2. As the primary endpoint of this study was safety and tolerability, the study achieved its primary endpoint. We also planned secondary exploratory endpoints to assess the impact of treatment on immune, and virologic endpoints, using samples obtained at leukapheresis before and following treatments.

### 3.1 Effects on immune parameters

Prespecified secondary endpoints were quantitative analyses on immune cell numbers and cytokine profiles, as well as plasma viremia and surrogate measures of reservoir size. CD4 T cell count in study subjects decreased significantly on study treatment irrespective of dose [baseline 783 cells/ mm^3^ (IQR 608, 1877) vs. week-24 724 cells/ mm^3^ (IQR 442, 948), p=0.003]. CD8 T cell count also decreased significantly in study subjects on study treatment [baseline 657 cells/ mm^3^ (IQR 329, 1402) vs. week-24 458 cells/ mm^3^ (IQR 246, 891), p=0.002,]. Differences in CD4 T cell and CD8 T cell counts persisted at week-28, four weeks after discontinuing study treatment (p=0.018 and p=0.026 respectively). CD4/CD8 ratio did not change significantly on study treatment. There were no significant differences in changes in CD4 T cell or CD8 T cell counts between dosing cohorts.

There were no significant changes in markers of activation (HLA-DR, CD38), proliferation Ki67, CD71) or exhaustion and immune checkpoints (CD73, CD160, PD1, Tim3, Lag3 or TIGIT) among peripheral CD4 or CD8 T cells on treatment across the dosing cohorts, when corrected for multiple comparisons (Fig. S2).

### 3.2 Effects on Inflammation

Multiplex cytokine measurement was performed in plasma samples obtained pre-treatment (Visit 1) and after the first cycle of study treatment (Visit 7). Ixazomib treatment did not induce a pro-inflammatory response in plasma (Fig S3). In unadjusted analyses, on-treatment plasma IL-6 and IL-12p40 concentrations were lower than pre-treatment (P=0.016 and P=0.008, respectively), although these differences were not statistically significant after correcting for multiple comparisons.

### 3.3 Effects on HIV viremia

**Fig. 2:**
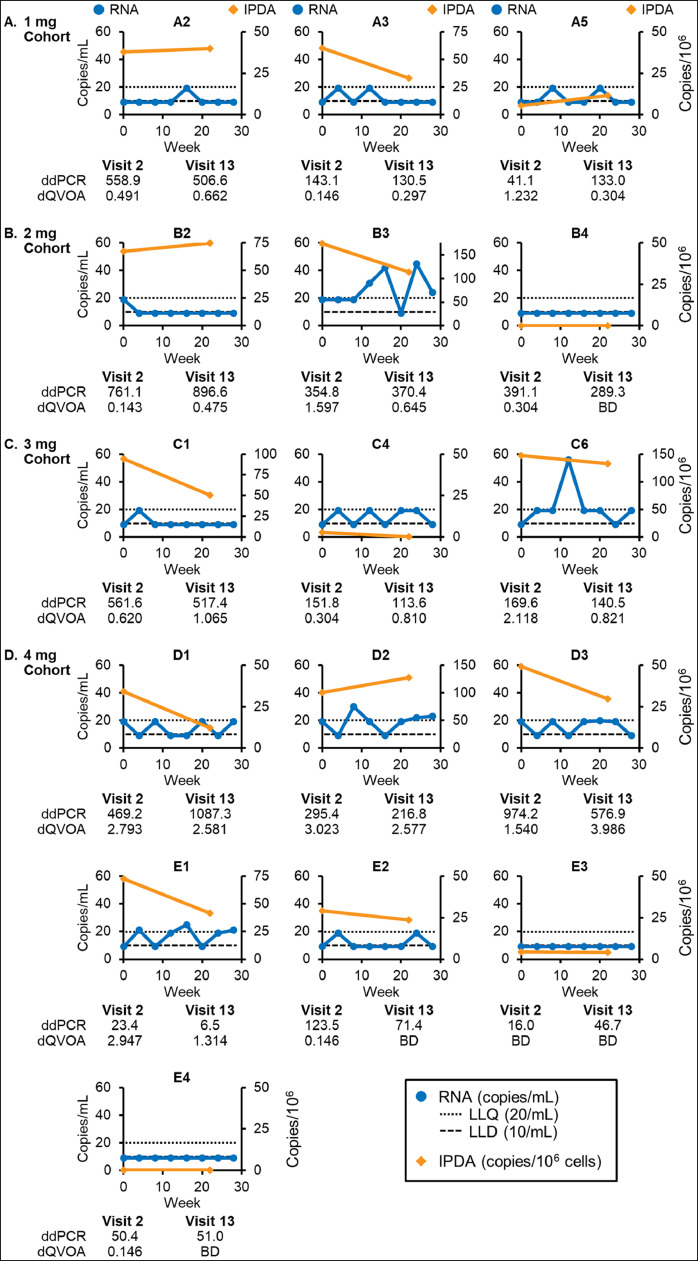
Ixazomib induced clinically measurable viral replication and reduction in peripheral HIV reservoir size in a subset of study participants. Plasma HIV viral load was measured in study participants before and during ixazomib treatment. Depicted are individual plasma HIV viral loads (left Y axis and blue lines) in the 4 dosing cohorts: 1 mg (A participants), 2 mg (B participants), 3 mg (C participants) and 4 mg (D and E participants). For clinical values of “Detected but <20”, the value is plotted at the LLQ minus 1. For clinical values of “Undetected”, the value is plotted at the LLD minus 1. LLQ – lower limit of quantification of 20 copies/ ml. LLD – lower limit of detection of 10 copies/ ml. Intact HIV proviral DNA measured by IPDA was measured in peripheral CD4 T cells (right Y axis, orange line). In addition, total cell associated HIV DNA was measured by ddPCR (pol copies/ 10^6^ CD4 T cells), and replication competent virus measured by dQVOA (infectious units per million CD4 T cells), at visits 2 and 13.

Despite having had continuously suppressed viral loads prior to study enrollment for several years, 4 (25%) participants experienced detectable plasma HIV viral load of greater than 20 copies/ ml on study treatment (Fig 2), in addition 9 other subjects experienced detectable viremia that was below the lower limit of quantitation. These included 1 participant treated with 2 mg dose (3 of 6 viral loads on-treatment detectable); 1 treated with 3 mg dose (1 of 6 viral loads on-treatment detectable) and 2 treated with 4 mg dose (both with 2 of 6 viral loads on-treatment detectable). The time to first viral blip on study medication ranged from 28 to 84 days. The maximum plasma HIV viral load on study treatment was 56 copies/ ml. There was no difference in pre-treatment duration of virologic suppression prior to study entry between those participants who experienced a blip compared to those that did not [median 515 days (IQR 356, 863) vs 1234 days (IQR 473, 2114), p=0.13, Mann Whitney test). These results are consistent with the ixazomib acting as a latency reversal agent, as we have described in vitro and ex vivo, [14] and as other have described with other proteasome inhibitors [13,15,24].

### 3.4 Effects on estimates of HIV reservoir size

HIV remains incurable in part because no interventions have yet been identified which reduce the size of the HIV reservoir in vivo. In part this is because accurate quantification of the HIV reservoir is difficult and requires very large numbers of cells to estimate the frequency of latently infected cells. To overcome that obstacle, we performed leukapheresis in all study subjects prior to receiving ixazomib, and between weeks 22 and 23 on ixazomib therapy. Because there is no consensus of which assay of reservoir size best reflects the latent reservoir, we used cryopreserved peripheral blood mononuclear cells (PBMCs) to assess change in HIV reservoir size from before to after study treatment, by multiple modalities (Supplementary Table S3).

**Fig. 3:**
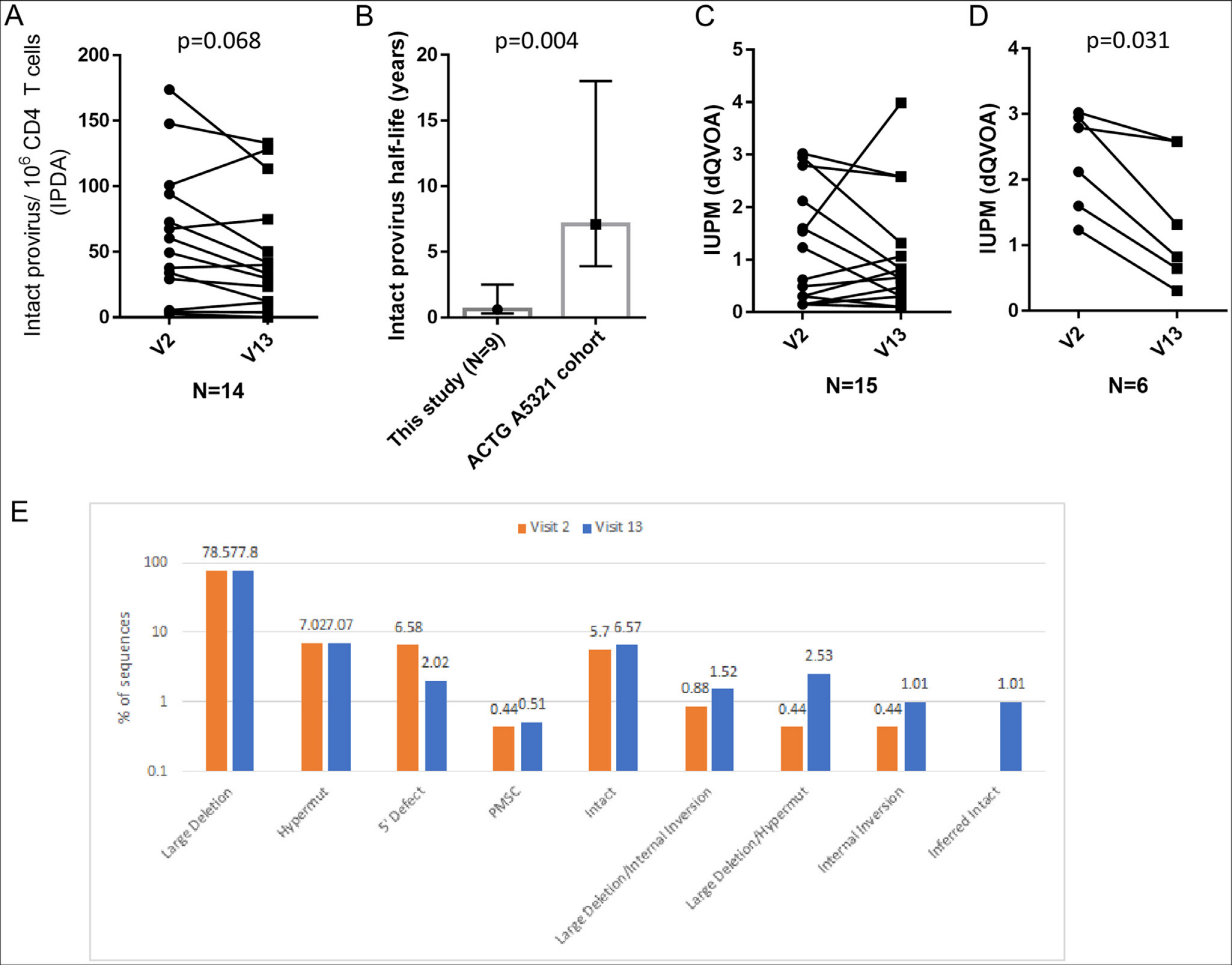
Ixazomib reduced peripheral HIV reservoir size in a subset of study participants. A) Intact HIV provirus was measured in circulating CD4 T cells obtained by leukapheresis before ixazomib treatment (Visit 2; V2) and during the last month of treatment (Visit 13; V3) by IPDA analysis. Depicted are data from 14 participants who had detectable intact HIV provirus at baseline. B) Intact provirus half life was estimated using nonlinear regression with one phase decay constrained with a plateau of 0 and compared to a previously published historical cohort. Only those participants who had two non-zero values (N=9) could have half life calculations. Error bars depict 95% confidence intervals. C) Replication competent latent provirus was estimated using dQVOA and expressed as infectious units per million resting CD4 T cells. Depicted are data from 15 participants who had detectable replication competent virus at baseline. D) IUPM estimates are depicted in a subset of participants as in (C) which had baseline IUPM values >1 and which completed the study protocol defined six months of ixazomib treatment. E) Proportions of intact and defective HIV proviral sequences obtained from circulating PBMCs at visits 2 and 13 are depicted (N=9 participants).

As expected, ixazomib treatment did not change total HIV-1 DNA levels in peripheral CD4 T cells as measured by ddPCR (Limit of detection >167 HIV-1 *pol* copies/ 10^6^ cells). Single genome, near full-length proviral sequencing in leukapheresis samples was performed in a subset of study participants (N=9). 426 HIV proviral species were analyzed in total, of which 108 (25%) near full length (8-9 kB) were subjected to next-generation sequencing, yielding 28 (6.5%) total genome-intact HIV-1 sequences. As this assay is not quantitative, comparison of numbers of genome-intact sequences could not be made. However, there were no significant differences in the composition of total HIV sequences in circulating PBMCs after ixazomib treatment in this subset of patients (Fig 3E).

The intact proviral DNA assay (IPDA) is a technical advance in estimating reservoir size as it is quantitative, more sensitive and able to differentiate intact versus non-intact HIV proviral DNA. Of the 16 participants that completed the study protocol, 14 (88%) had intact HIV proviruses detected by IPDA in peripheral CD4 T cells at baseline. Intact provirus level decreased numerically on ixazomib treatment in 10/14 (71%), (median difference -10.2 copies/ 10^6^ cells, p=0.068, Wilcoxon matched-pairs signed rank test) (Fig 3A). This result allowed calculation of an estimated half life of intact provirus in that subset of patients. The median estimated half life of intact provirus for those participants treated with ixazomib was 0.6 years (95% CI 0.3, 2.5), significantly shorter than what has been previously reported in ART-suppressed patients using the same assay, performed in the same lab (7.1 years (95% CI 3.9, 18), p=0.004, one sample Wilcoxon Signed Rank Test)(Fig 3B) [35].

A third, less sensitive but highly specific measure of latent proviruses are quantitative viral outgrowth assays, which measures replication competent and inducible provirus. dQVOA uses ex vivo differentiation of resting CD4 T cells to enhance HIV reactivation, outgrowth, and assay sensitivity compared to standard QVOA measurements on virologically suppressed HIV positive persons [27]. Of the 16 study participants, 15 (94%) had measurable proviruses detected by dQVOA at baseline. dQVOA results declined in 9/15 subjects at end of study. However, the overall cohort change was not significant (median difference -0.05 IUPM (infectious units per million CD4 T cells, p=0.48, Wilcoxon matched-pairs signed rank test) (Fig 3C). In the subset of participants (N=6) that had a baseline IUPM>1, and who completed six cycles of ixazomib treatment per protocol, ixazomib treatment significantly reduced the replication competent viral reservoir (median difference -0.94 IUPM, p=0.031, Wilcoxon matched-pairs signed rank test) (Fig 3D).

## 4 Discussion

Combination ART treatment has remarkably reduced morbidity and mortality in those HIV infected individuals who can access therapy; however, lifelong ART is required because current treatments do not clear the HIV-1 reservoir. The HIV reservoir is established early during primary HIV-1 infection [36]. After initiation of continuous ART, there is a rapid but incomplete decay of HIV-1 reservoir size [37], followed by a period of much slower decay with an estimated half life of 44 months [38] that persists for more than decade on therapy making viral eradication within an individual's lifetime unachievable with only 2 known exceptions.

That recognition has led to preclinical and early clinical assessment of host directed therapies which may offer additional therapeutic potential. Considerable precedent exists for targeting host pathways that are activated by viruses, for example neutralizing inflammatory mediators which contribute to pathogenesis (e.g. IL-6 inhibitors for COVID-19), enhancing the immune response against the virus (e.g. interferon treatment of Hepatitis C) and antagonizing host pathways required for viral persistence (e.g. birinapant for Hepatitis B). In the setting of HIV, host directed therapies are under evaluation to achieve latency reversal, latency silencing, immune augmentation, clonal expansion or inhibition of clonal expansion of the reservoir, in addition to gene editing [39]. Despite preclinical promise, to date none of these approaches have caused a meaningful reduction in HIV reservoir size in HIV positive individuals.

We have previously shown that the clinically relevant proteasome inhibitors ixazomib and bortezomib increase cell death of HIV infected over uninfected cells *in vitro* [14]. Furthermore, using HIV-LTR reporter constructs, we have described that ixazomib directly induces HIV replication in an NFkB dependent manner [14], similar to the reports of bortezomib and carfilzomib causing latency reversal and increased expression of HIV RNA in both cell lines and ex vivo cells from HIV positive donors [13,15,24]. Given these preclinical findings that ixazomib both reactivates HIV and promotes the killing of HIV infected more than uninfected cells, we opted to test ixazomib in the first open label phase 1b/2a clinical trial of this agent in HIV positive participants. Ixazomib was selected over bortezomib due to its better clinical safety profile and oral route of administration. The dose-escalation, uncontrolled study design was conceived with the direction of the FDA as part of the IND application.

The primary outcome of our phase 1b/2a clinical trial was to determine the safety and tolerability of oral ixazomib in ART-suppressed, HIV positive persons. To that end, ixazomib treatment was well tolerated with no grade 2 or higher adverse effects, and the maximum tolerated dose was determined to be the maximum dose tested, 4 mg weekly. As this study was designed as an open label, dose escalating design, it is not powered to detect treatment emergent effects, and so no definitive conclusions are possible in that regard. However, we designed predefined exploratory secondary analyses to determine if any potential signals of latency reversal or anti-reservoir effect are seen in vivo. Participants experienced statistically significant, but not clinically meaningful, reductions in peripheral absolute CD4 T cell counts and CD8 T cell counts, but not in CD4/ CD8 ratios. This was likely due to mild drug induced lymphopenia, which is noted in clinical use of ixazomib in the setting of multiple myeloma. Notably, 4 of the 16 participants that completed the trial experienced clinically detectable viral reactivation, as measured by plasma HIV viral loads >20 copies/ ml (Fig 2), and another 9/16 had detectable viremia that was below LLQ, which by comparison with viral load measures of the same subjects over the preceding 24 weeks (Fig S4), is suggestive of and consistent with *in vivo* HIV latency reversal by ixazomib. Also, of note, low level viremia did not come at the cost of excessive immune activation, proliferation marker expression, exhaustion, or immune checkpoint marker expression as assessed by flow cytometry of CD4 and CD8 T cell subsets or measurement of pro-inflammatory cytokines in the plasma (Fig S2 and Fig S3).

While other clinical interventions have achieved limited viral reactivation following in vivo treatment, none have achieved reductions in surrogate measures of reservoir size [8,10,40,41]. We measured changes in HIV reservoir size using peripheral blood cells, obtained by leukapheresis, before and after study treatment by multiple methods. No differences were seen when reservoir size was estimated using ddPCR or viral sequencing. Arguably, the most relevant quantitative measures of reservoir estimation are IPDA and dQVOA, and in those assays 5 patients (subjects B3,C6,D1,E1,E2) had declines in reservoir size by both QVOA and IPDA, and another 5 (subjects A5,B4,C1,D2,E4) had reductions in one or other measure. Also, not all study participants received maximum tolerated dose of ixazomib; of the seven study subjects that did, 5/7 had reductions in dQVOA while three had reductions in IPDA, suggesting that at the maximum dose tested there is a possible enhanced anti-reservoir effect. Considering the short duration of this trial (6 months), and the long half life of the latent viral reservoir (typically estimated in years), our data indicate that ixazomib treatment likely accelerated the decay in HIV reservoir size in at least a subset of participants (0.6 years in this study, compared to 7.1 years in similar patients using the same assay, performed in the same lab, Figure 3B). Definitive inferences on the effects of ixazomib on HIV reservoir size, though, are limited by the small number of participants included in this early phase trial, the lack of an untreated control group, and the short duration of the trial. Therefore, future trials of ixazomib should consider using the maximum tolerated dose, should be powered for anti-reservoir efficacy, and consider longer periods of treatment based on data in myeloma patients showing that treatment over years remains well tolerated [42].

Our data suggest that ixazomib treatment is associated with reductions in HIV reservoir size in HIV infected persons. Moreover, it is notable that this treatment is simple – oral and once weekly, safe in this cohort of patients, does not have any known drug interactions with first line antiretroviral agents, and is therefore potentially scalable within an ambulatory care model. Much like combination of antivirals have proven to be superior to single agents for treatment of chronic viral infection, and combinations of cancer agents are superior to single agents for cure of malignancies, it is likely that combinations of agents active against the HIV reservoir will be required to achieve the goal of HIV cure. These data suggest that ixazomib is one agent that is active against the HIV reservoir and may represent the first step in the journey towards an HIV cure and testing its use in controlled clinical trials coupled with intensive mechanistic studies is warranted.

## Funding

This study was funded by Millennium Pharmaceuticals Inc..; the Mayo Clinic Foundation; the National Institutes of Health, including the National Institute of Allergy and Infectious Diseases, Division of AIDS, the National Heart, Lung and Blood Institute, the National Institute of Diabetes and Digestive and Kidney Diseases, the National Institute of Neurological Disorders and Stroke, and the National Institute on Drug Abuse. Mayo Clinic also acknowledges generous funding support from Mr. Joseph T. and Mrs. Michele P. Betten.

## Data sharing statement

All data is available in the main text or the supplementary materials.

## Declaration of Competing Interest

NWC was supported by R56 AI145407-01A. ADB was supported by R01 AI110173, R01 AI120698; Amfar (#109593). ML is supported by NIH grants AI130005, AI117841, AI152979, DK120387, DA047034, AI120008. RC is supported by NIH grants 1U01AI131566-0, 1R01HD097843-01,1R21HD103498-01. This project was conducted in part by Southern Research using federal funds from the Division of AIDS, National Institute of Allergy and Infectious Diseases, National Institutes of Health under contract HHSN272201500017C entitled “Quantitative Viral Outgrowth Assay (QVOA) Service Resource. This work was also supported by UM1AI164562, co-funded by National Heart, Lung and Blood Institute, National Institute of Diabetes and Digestive and Kidney Diseases, National Institute of Neurological Disorders and Stroke, National Institute on Drug Abuse and the National Institute of Allergy and Infectious Diseases. ADB is a paid consultant for Abbvie, Gilead, Freedom Tunnel, Pinetree therapeutics Primmune, Immunome, MarPam, and Flambeau Diagnostics, is a paid member of the DSMB for Corvus Pharmaceuticals, Equilium, and Excision Biotherapeutics, has received fees for speaking for Reach MD and Medscape, owns equity for scientific advisory work in Zentalis and Nference, and is founder and President of Splissen therapeutics. Mayo Clinic has filed a patent on the use of ixazomib in HIV positive persons. GML is an employee of and owns equity in Accelevir Diagnostics. NWC received funding from Takeda (Millennium Pharmaceuticals Inc) to conduct the trial, including professional time. AJT receives honoraria from Uptodate.com for writing unrelated to this topic. JZ serves on the advisory board for ViiV. SK receives research support for clinical trials from Abbvie, Celgene, Janssen, Takeda, Adapative, KITE, Medimmune/ Astra Zeneca, Merck, Novartis, Roche and Sanofi. SK is an advisory board member for Abbvie, Celgene, Janssen, Takeda, Adaptive, KITE, and Medimmune/ Astra Zeneca. SK participates in an independent review committee for Oncopeptides.

## Appendix Supplementary materials

Image, application 1
