# Supplementary material for: Single center, open label dose escalating trial evaluating once weekly oral ixazomib in ART-suppressed, HIV positive adults and effects on HIV reservoir size in vivo
Source: eClinicalMedicine. 2021 Nov 29;42:101225. doi: 10.1016/j.eclinm.2021.101225 (PMC8639424; doi:10.1016/j.eclinm.2021.101225)
Supplement: Supplementary file 1 [file mmc1.docx]

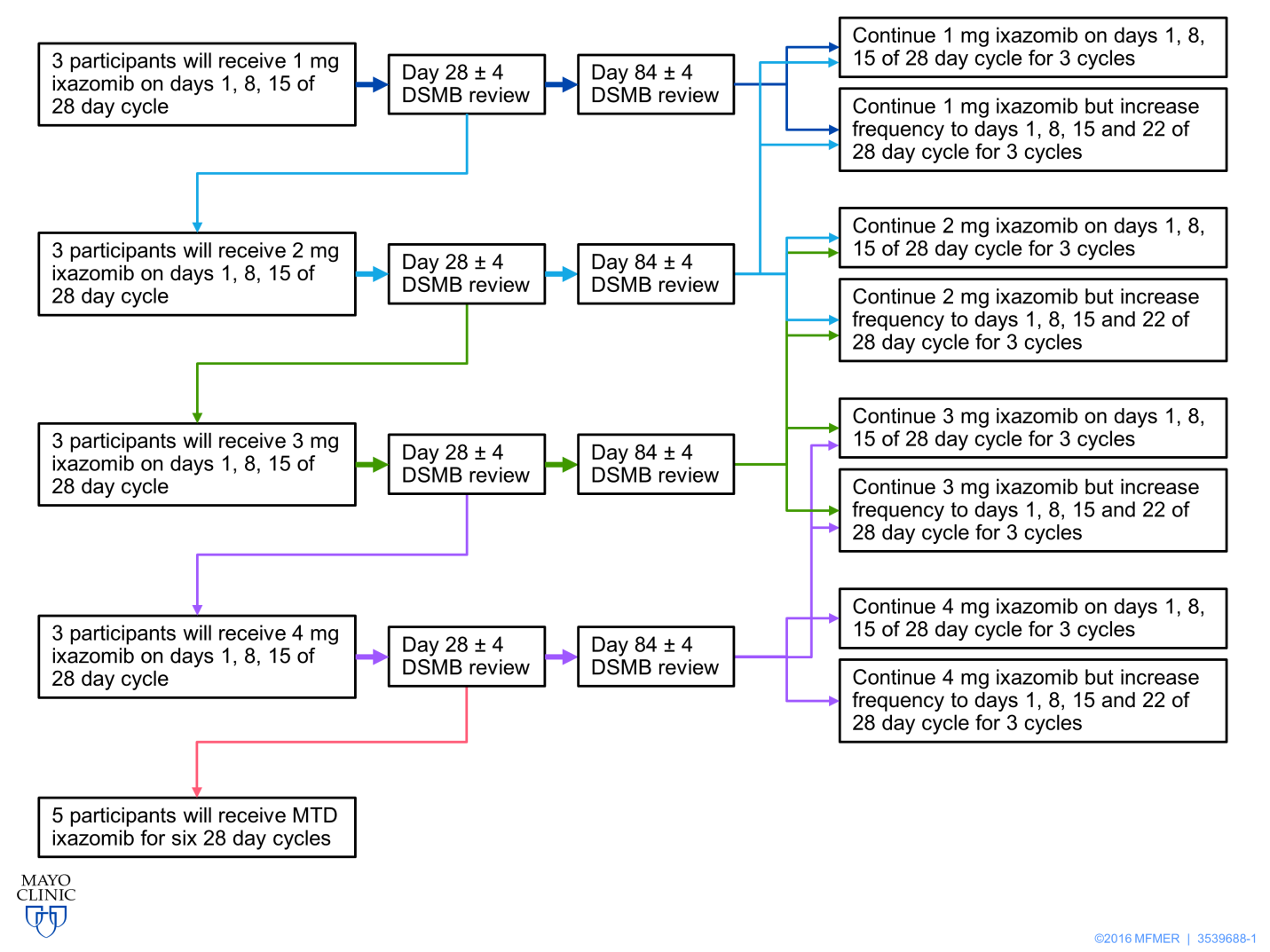
Fig. S1.

Per protocol study design. DSMB – Data Safety Monitoring Board. MTD – Maximum tolerated dose.


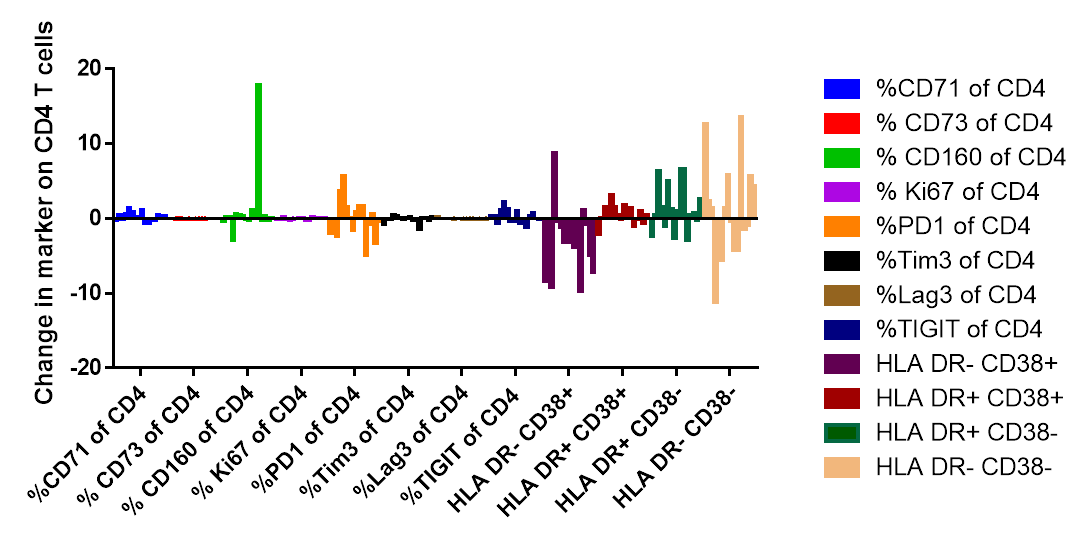


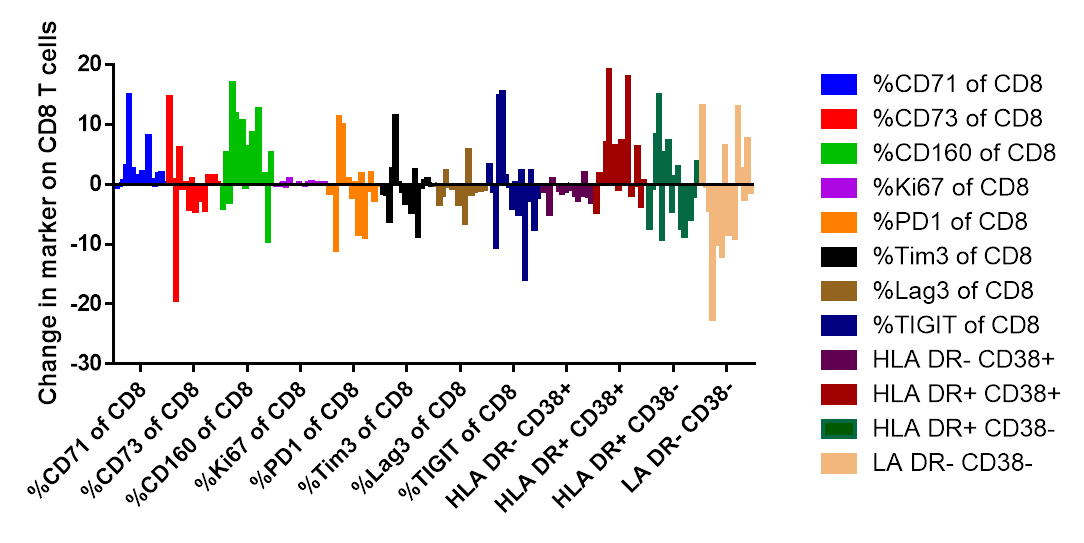


Fig. S2.

Depicted are individual absolute changes in percentage of CD4 T cells (top panel) or CD8 T cells (bottom panel) expressing the noted markers of T cell activation or exhaustion. No changes were statistically significant after correction for multiple comparisons.


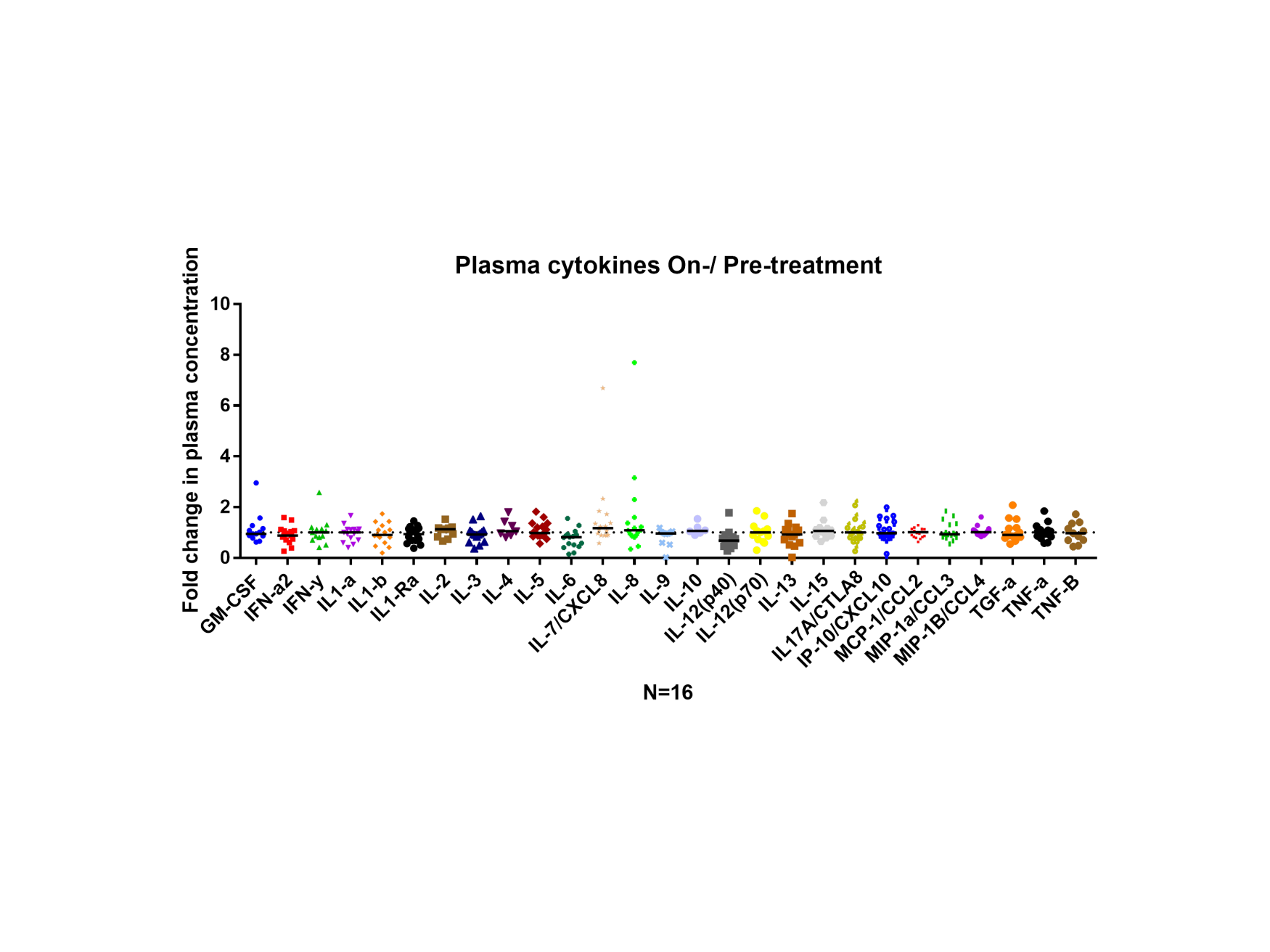


Fig. S3.

Depicted are individual relative changes in plasma concentrations of the noted cytokines comparing pre-treatment values to after the first cycle of study treatment (Study Visit 7). No changes were statistically significant after correction for multiple comparisons.


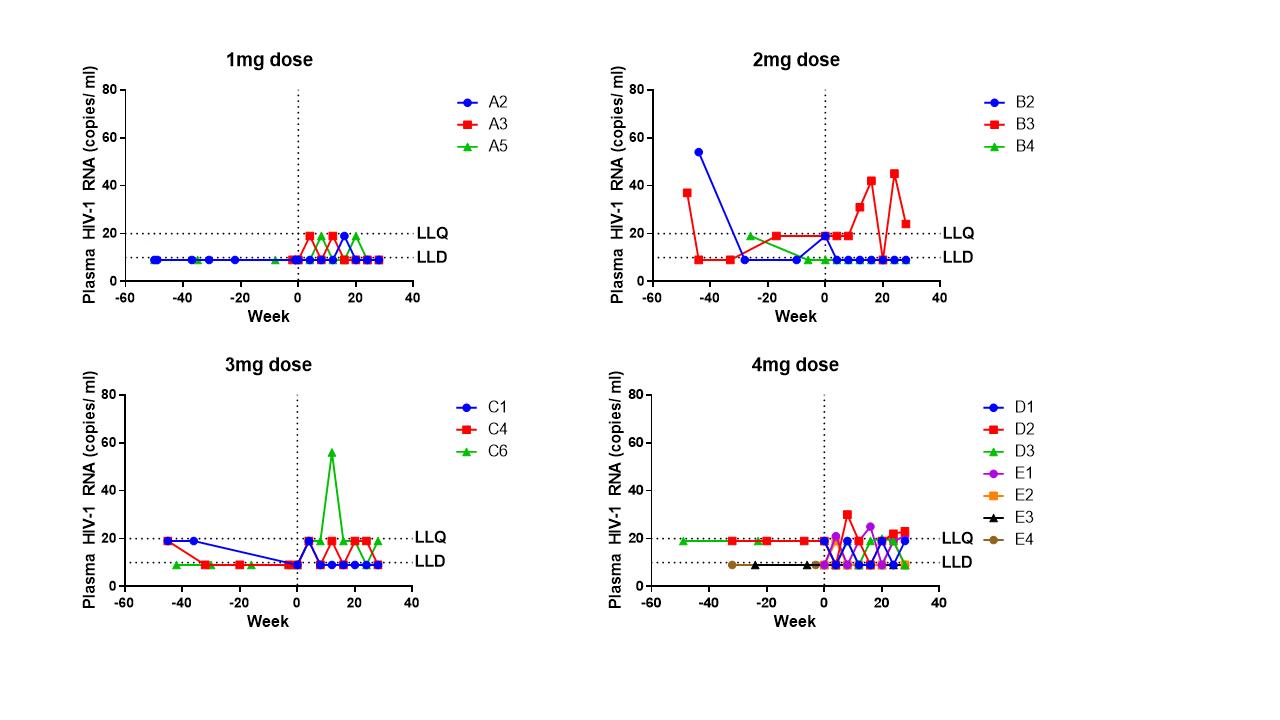


Fig. S4.

Depicted are individual plasma concentrations of HIV RNA viral load comparing pre-trial values (left of vertical dashed line) to on study values (right of vertical dashed line). LLQ – lower limit of quantification. LLD – lower limit of detection.

Table S1.

Baseline characteristics of the study participants (N=17).

| **Cohort** | **A, 1 mg (N=4)** | **B, 2 mg (N=3)** | **C, 3mg (N=3)** | **D&E, 4mg (N=7)** |
| --- | --- | --- | --- | --- |
| **Age, median**  **(Q1, Q3)** | 54.1  (49.9, 58.2) | 49.3  (40.9, 52.3) | 51.0  (39.3, 53.5) | 49.0  (41.2, 52.0) |
| **Gender**  **Male**  **Female** | 4 (100%)  0 (0.0%) | 3 (100%)  0 (0.0%) | 3 (100%)  0 (0.0%) | 6 (85.7%)  1 (14.3%) |
| **CD4 count**  **(cells/mmˆ(3))**  **Median (Q1, Q3)** | 724 (676, 830) | 914 (790, 968) | 1130 (1029,  1240) | 735 (680, 770) |
| **CD8 count**  **(cells/mmˆ(3))**  **Median (Q1, Q3)** | 508 (504, 810) | 803 (716, 834) | 1014 (868,  1208) | 573 (405, 712) |
| **CD4/CD8 Ratio**  **Median (Q1, Q3)** | 1.45 (1.10,  1.67) | 1.14 (0.95,  1.38) | 1.28 (1.04,  1.31) | 1.58 (1.15,  1.82) |

Table S2.

Adverse Events and Side Effects.

|  | 1 mg (N=4) | 2 mg (N=3) | 3 mg (N=3) | 4 mg (N=7) | Total (N=17) |
| --- | --- | --- | --- | --- | --- |
| Cognitive disturbance | 0 (0.0%) | 0 (0.0%) | 0 (0.0%) | 0 (0.0%) | 0 (0.0%) |
| Constipation | 1 (25.0%) | 0 (0.0%) | 0 (0.0%) | 0 (0.0%) | 1 (5.9%) |
| Diarrhea | 1 (25.0%) | 0 (0.0%) | 1 (33.3%) | 1 (14.3%) | 3 (17.6%) |
| Edema Limbs | 0 (0.0%) | 1 (33.3%) | 0 (0.0%) | 0 (0.0%) | 1 (5.9%) |
| Fatigue | 0 (0.0%) | 0 (0.0%) | 1 (33.3%) | 0 (0.0%) | 1 (5.9%) |
| Febrile neutropenia | 0 (0.0%) | 0 (0.0%) | 0 (0.0%) | 0 (0.0%) | 0 (0.0%) |
| Nausea | 0 (0.0%) | 0 (0.0%) | 1 (33.3%) | 0 (0.0%) | 1 (5.9%) |
| Peripheral motor neuropathy | 0 (0.0%) | 0 (0.0%) | 1 (33.3%) | 0 (0.0%) | 1 (5.9%) |
| Peripheral sensory neuropathy | 0 (0.0%) | 0 (0.0%) | 1 (33.3%) | 0 (0.0%) | 1 (5.9%) |
| Rash, maculopapular | 0 (0.0%) | 1 (33.3%) | 0 (0.0%) | 1 (14.3%) | 2 (11.8%) |
| Sepsis | 0 (0.0%) | 0 (0.0%) | 0 (0.0%) | 0 (0.0%) | 0 (0.0%) |
| Vomiting | 0 (0.0%) | 0 (0.0%) | 0 (0.0%) | 0 (0.0%) | 0 (0.0%) |

**Table S3.** Peripheral HIV reservoir size measured before (Visit 2) and near the end of study treatment (Visit 13).

|  |  | **ddPCR** | **IPDA** |  |  |  | **dQVOA** |  |  |
| --- | --- | --- | --- | --- | --- | --- | --- | --- | --- |
| **ID** | **Visit Number** | **Pol copies per** | **Count Per Million CD4 T cells** | | | | **IUPM in rCD4 T cells** | | |
|  |  | **Million CD4 T cells** | **Intact** | **3' Defective** | **5' Defective** | **Total Proviruses Detected** | **IUPM** | **lobound** | **hibound** |
| **A2** | 2 | 558.9 | 37.74 | 540.56 | 259.01 | 837.31 | 0.491 | 0.158 | 1.525 |
| **A2** | 13 | 506.6 | 40.06 | 504.25 | 267.70 | 812.02 | 0.662 | 0.247 | 1.772 |
| **A3** | 2 | BD (167.0) | 60.48 | 213.94 | 103.71 | 378.13 | 0.146 | 0.021 | 1.038 |
| **A3** | 13 | BD (167.0) | 33.18 | 242.93 | 117.95 | 394.06 | 0.297 | 0.074 | 1.188 |
| **A5** | 2 | BD (167.0) | 5.34 | 130.08 | 72.45 | 207.87 | 1.232 | 0.583 | 2.604 |
| **A5** | 13 | BD (167.0) | 11.61 | 78.91 | 46.76 | 137.29 | 0.304 | 0.076 | 1.216 |
| **B2** | 2 | 761.1 | 67.62 | 823.20 | 494.20 | 1385.03 | 0.143 | 0.020 | 1.015 |
| **B2** | 13 | 896.6 | 74.99 | 1012.50 | 627.20 | 1714.69 | 0.475 | 0.153 | 1.477 |
| **B3** | 2 | 354.8 | 173.72 | 259.08 | 192.72 | 625.52 | 1.597 | 0.785 | 3.246 |
| **B3** | 13 | 370.4 | 113.42 | 188.60 | 122.81 | 424.82 | 0.645 | 0.241 | 1.724 |
| **B4** | 2 | 391.1 | - | - | 54.24 | 54.24 | 0.304 | 0.076 | 1.216 |
| **B4** | 13 | 289.3 | - | - | 45.00 | 45.00 | BD (0.098) | 0.000 | 0.422 |
| **C1** | 2 | 561.6 | 94.25 | 413.62 | 156.97 | 664.83 | 0.620 | 0.232 | 1.656 |
| **C1** | 13 | 517.4 | 50.26 | 432.33 | 141.29 | 623.87 | 1.065 | 0.475 | 2.391 |
| **C4** | 2 | BD (167.0) | - | - | 129.73 | 129.73 | 0.304 | 0.076 | 1.216 |
| **C4** | 13 | BD (167.0) | - | - | 98.48 | 98.48 | 0.810 | 0.336 | 1.954 |
| **C6** | 2 | 169.6 | 147.70 | 454.32 | 412.34 | 1014.36 | 2.118 | 1.115 | 4.022 |
| **C6** | 13 | BD (167.0) | 132.98 | 558.18 | 434.10 | 1125.26 | 0.821 | 0.340 | 1.981 |
| **D1** | 2 | 469.2 | 34.08 | 104.47 | 214.52 | 353.07 | 2.793 | 1.536 | 5.079 |
| **D1** | 13 | 1087.3 | 12.23 | 147.85 | 331.58 | 491.67 | 2.581 | 1.381 | 4.823 |
| **D2** | 2 | 295.4 | 100.78 | 134.11 | 181.41 | 416.29 | 3.023 | 1.645 | 5.555 |
| **D2** | 13 | 216.8 | 128.03 | 125.77 | 197.90 | 451.69 | 2.577 | 1.375 | 4.828 |
| **D3** | 2 | 974.2 | 49.36 | 433.03 | 604.10 | 1086.49 | 1.540 | 0.760 | 3.121 |
| **D3** | 13 | 576.9 | 29.76 | 352.81 | 463.88 | 846.45 | 3.986 | 2.137 | 7.436 |
| **E1** | 2 | BD (167.0) | 72.74 | 417.79 | 497.53 | 988.06 | 2.947 | 1.608 | 5.399 |
| **E1** | 13 | BD (167.0) | 41.77 | 375.31 | 481.37 | 898.45 | 1.314 | 0.619 | 2.788 |
| **E2** | 2 | BD (167.0) | 29.18 | 188.16 | 286.67 | 504.02 | 0.146 | 0.021 | 1.038 |
| **E2** | 13 | BD (167.0) | 23.56 | 189.75 | 268.12 | 481.44 | BD (0.098) | 0.000 | 0.422 |
| **E3** | 2 | BD (167.0) | 4.27 | 20.89 | 13.49 | 38.65 | BD (0.098) | 0.000 | 0.422 |
| **E3** | 13 | BD (167.0) | 3.97 | 13.71 | 6.63 | 24.31 | BD (0.098) | 0.000 | 0.422 |
| **E4** | 2 | BD (167.0) | BD | 4.84 | BD | 4.84 | 0.146 | 0.021 | 1.038 |
| **E4** | 13 | BD (167.0) | BD | 11.38 | BD | 11.38 | BD (0.098) | 0.000 | 0.422 |

*ddPCR – digital droplet PCR; IPDA – intact proviral DNA assay; dQVOA – differentiation quantitative viral outgrowth assay; rCD4 – resting CD4; BD – below limit of detection; lobound – low boundary of estimate; hibound – high boundary of estimate.
